# Role of positive mental health in reducing fears related to COVID-19 and general anxiety disorder in Khyber Pakhtunkhwa, Pakistan

**DOI:** 10.1186/s40359-022-00869-7

**Published:** 2022-06-27

**Authors:** Qaisar Khalid Mahmood, Malik Muhammad Sohail, Waheed Ahmad Qureshi, Rubeena Zakar, Kamil J. Wrona, Florian Fischer

**Affiliations:** 1 Department of Gender Studies, University of the Punjab, Lahore, Pakistan; 2 Department of Sociology, University of Chakwal, Punjab, Pakistan; 3 Department of Sociology, Anadolu Üniversitesi Yeşiltepe, Eskişehir, Turkey; 4 Institute of Social and Cultural Studies, University of the Punjab, Lahore, Pakistan; 5 School of Public Health, Bielefeld University, Bielefeld, Germany; 6 Institute of Public Health, Charité – Universitätsmedizin Berlin, Berlin, Germany; 7 Bavarian Research Center for Digital Health and Social Care, Kempten University of Applied Sciences, Kempten, Germany

**Keywords:** COVID-19, SARS-CoV-2, Corona, Pakistan, Mental health, Quality of life

## Abstract

**Background:**

The outbreak of the novel coronavirus disease (COVID-19) has posed multiple challenges to healthcare systems. Evidence suggests that mental well-being is badly affected due to compliance with preventative measures in containing the COVID-19 pandemic. This study aims to explore the role of positive mental health (subjective sense of wellbeing) to cope with fears related to COVID-19 and general anxiety disorder in the Pashtun community in Pakistan.

**Methods:**

A cross-sectional survey was conducted among 501 respondents from Khyber Pakhtunkhwa participating in an online-based study. We performed correlational analysis, hierarchical linear regression and structural equational modeling (SEM) to analyze the role of mental health in reducing fears and general anxiety disorder.

**Results:**

The results of the SEM show that positive mental health has direct effects in reducing the fear related to COVID-19 (β = − 0.244, p < 0.001) and general anxiety (β = − 0.210, p < 0.001). Fears of COVID-19 has a direct effect on increasing general anxiety (β = 0.480). In addition, positive mental health also has an indirect effect (β = − 0.117, p < 0.001) on general anxiety (R^2^ = 0.32, p < 0.001) through reducing fear of coronavirus.

**Conclusion:**

Based on these findings, there is a need to develop community health policies emphasizing on promotive and preventive mental health strategies for people practicing social/physical distancing.

## Background

The COVID-19 pandemic has generated a wide range of emotions, thoughts and reactions worldwide [1]. From the beginning, it has shown a rapid increase in the mortality rate, creating unprecedented fears among the public [2]. Observing an extremely rapid infection and high mortality rate—along with an ‘infodemic’—made people extremely worried about COVID-19 [3, 4]. Undoubtedly, the media has played an important role in encouraging preventive behavior [5]. However, excessive media reporting has also created an extremely stressful situation. Such a large-scale outbreak may be followed by panic, fear, suspicion and stigma [6]. In the wake of this pandemic, mental health has become an emergent global challenge. Psychological distress and anxiety have been experienced by survivors during epidemics in recent years [7–9]. Similar experiences of mental health disorders—such as obsession-compulsion, anxiety, hostility, phobic anxiety, paranoid ideation and psycho-trauma—were shared either by survivors of Ebola and Zika outbreaks in Africa [10–12].

Fear of contracting the COVID-19 infection has been observed among people worldwide [13, 14]. In such a situation, this fear may increase the intensity of the disease itself [15]. During such a large-scale health crisis, individuals may not make rational decisions regarding behavior changes in order to evade any viral contagion [16]. The World Health Organization (WHO) has highlighted a substantial need for psychological interventions during the COVID-19 outbreak to avoid any further crisis of compromised mental health for those suffering from the infection itself, and for those who are in self-isolation or quarantined [17].

Until a couple of decades ago, the focus of mental health professionals was largely on negative mental health issues such as anxiety, depression, delusions, phobias and obsessions. The prime emphasize was on preventing mental health problems. However, this perspective neglects the idea of a fulfilled individual and a thriving community. Conversely, positive mental health is more focusing on an individual’s functioning, quality of life, and wellbeing [18, 19]. In recent years, new evidence-based psychology has emerged which pays attention to positive emotions and positive mental health [20]. Many recent studies emphasize the significance of positive emotions in mental health and well-being [21].

Positive mental health revolves around positive emotions, feelings and functioning, which help individuals cope with the normal stresses of life, work productively and fruitfully, and to contribute towards the improvement of community life by using their own abilities [22]. Its conceptualization may consist of the presence of multiple human strengths, the dominance of positive emotions, high socio-emotional intelligence, subjective well-being and resilience [18]. Increasing evidence suggests that high levels of positive mental health reduce the propensity of mental illness [23]. It may help to prevent and treat psychopathology [24].

Globally, a plethora of studies [25–34] have focused on negative mental health conditions caused by the COVID-19 pandemic. Until now, studies focusing on positive mental health to cope with COVID-19 related anxiety, fears and stress have rarely been found. The present study aims to investigate the role of positive mental health in reducing fears and anxiety during the COVID-19 pandemic. Based on the literature, we hypothesize that people who use positive mental health strategies are more likely to handle their fears and anxiety during the pandemic adequately.

## Methods

### Study setting

With a population of 35.5 million, Khyber Pakhtunkhwa is the third largest province of Pakistan; it is located in the northwestern region of the country [35]. Even after getting the provincial autonomy, the healthcare system of Khyber Pakhtunkhwa is in the process of development. Therefore, the region is unable to endure the influx of patients in case of medical emergency [36], which is visible in high numbers of COVID-19 cases and deaths [37]. An increasing number of COVID-19 cases can exacerbate psychological challenges and complications for the population. Ravaged by military operation, terrorist attacks and population displacement within the last two decades, the Khyber Pakhtunkhwa province only has few mental health facilities.

### Study design and data collection

A cross-sectional study was conducted in the general population of Khyber Pakhtunkhwa, Pakistan. At time of data collection, a nationwide lockdown was implemented in Pakistan. Therefore, we conducted this study using an online-based survey. For this purpose, we created a questionnaire on Google Survey and the hyperlink was shared among social media users of Khyber Pakhtunkhwa through various social media platforms such as Facebook, LinkedIn and WhatsApp. We collected data over a two-week period from May 10 to May 23, 2020. There was no specific reason of restricting data collection to this a respective time phrase, but we aimed to complete data during a comparatively short time due to the fast and unpredictable course of the pandemic. The survey was successfully completed by 501 participants.

### Measuring instruments

#### Positive mental health

The ‘Positive Mental Health Scale’ (PMH-scale) [38] was used to assess the holistic concept of positive emotionality related to positive mental health. This scale consists of nine items (e.g. “I am often carefree and in good spirits”, “I manage well to fulfill my needs”, and “I feel that I am actually well-equipped to deal with life and its difficulties”). These items were rated on a Likert scale from 1 (not true) to 4 (true). The reported value of Cronbach’s alpha was 0.842 indicating good internal consistency.

#### Fear of COVID-19

Recently, the ‘Fear of COVID-19 Scale’ (FCV-19S) has been developed to assess the fear of COVID-19 [15]. The FCV-19S is a 7-item scale (e.g. “It makes me uncomfortable to think about Corona” and “I cannot sleep because I worry about getting Corona”) measured on a 5-point Likert scale. A five-point Likert-scale (1 = “strongly disagree” to 5 = “strongly agree”) is used to report the responses of the respondents [15]. This scale has been developed in English language, but has been validated in other languages as well [39–41]. Mahmood and his colleagues have validated the Urdu version of FCV-19S in Pakistan [42]. The scale was reliable, indicated by Cronbach’s alpha value of 0.872.

#### Preventive behavior related to COVID-19

According to available knowledge regarding COVID-19 and the recommendations provided by the WHO [17], Mahmood et al. [42] developed seven statements to measure preventive behavior related to COVID-19 (e.g. “I regularly wash my hands for twenty seconds” and “I maintain social/physical distancing while meeting others”). These items were measured on a 5-point Likert scale (1 = “strongly disagree” to 5 = “strongly agree”). The scale was highly reliable (α = 0.846).

#### Generalized anxiety disorder

The Generalized Anxiety Disorder (GAD) questionnaire was developed to measure anxiety disorders [43]. This scale consists of seven items. These items describe a number of the most salient diagnostic features of GAD (i.e., feeling nervous, anxious, or on edge, and worrying too much about various things). Items are rated on a 4-point Likert-type scale (1 = “not at all” to 4 = “almost every day”) with high reliability (α = 0.892). The distribution of scales can be considered normal if the values of skewness and kurtosis range until + 2.0 [44]. The results show that all the scales were normally distributed, except for general anxiety disorder, where the value for skewness was higher than 2.0.

### Statistical analysis

All analyses were conducted with IBM SPSS Statistics 21. Descriptive statistics were used to report the sample characteristics. Measures of central tendency (mean and standard deviation [SD]) and measures of distribution (skewness and kurtosis) were calculated with respect to each item. Cronbach’s alpha coefficient (α) and correlation matrix were calculated. We performed a multiple linear regression model by using a hierarchal method to analyze factors contributing to general anxiety disorder. Finally, path analysis via structural equational modelling (SEM) was conducted. AMOS software was used for this purpose. Goodness of fit was assessed according to the following criteria: goodness of fit index (GFI > 0.90), comparative fit index (CFI > 0.90), root mean square residual (RMSR < 0.08), and root mean square error of approximation (RMSEA < 0.08). Direct and indirect effects were also calculated.

## Results

### Descriptive analyses

Out of 501 respondents, more than half (58.5%) were male and approximately 61% living in urban areas. Most participants (54.5%) belonged to the 26–50 years age group, followed by 38.9% who were aged up to 25 years. Most of the respondents were either graduates (41.5%) or postgraduates (46.9%). The study population had an almost equal representation of unmarried and married people, and more than half of the respondents (56.7%) were unemployed. There was no significant mean difference in fear of COVID-19 related to the living area. However, married people had more fear as compared to unmarried people.

In Table 1, the measures of central tendency and distribution for study variables are presented. The findings show that the respondents had high average scores of positive mental health and preventive behavior. In addition, they had moderate average scores of fears of COVID-19 and general anxiety disorder.

**Table 1:** Psychometric properties of study variables (n = 501)

Variables	Number of items	Mean	SD	Kurtosis	Skewness	Cronbach’s alpha
Positive mental health	9	34.98	4.66	− 0.184	0.857	0.842
Fear of COVID-19	7	18.57	5.572	0.251	0.066	0.872
Preventive behavior	7	27.16	6.021	− 0.801	− 0.002	0.846
General anxiety disorder	7	10.33	4.618	1.752	2.791	0.892

SD, standard deviation

### Bivariate analysis

A correlational analysis (Table 2) was conducted to observe the relationships among study variables. The findings show that positive mental health has a negative and significant relationship with fear of COVID-19 (r = − 0.228, p < 0.01) and general anxiety disorder (r = − 0.277, p < 0.01). Positive mental health also has a positive and significant association with preventive behavior (r = 0.122, p < 0.01). In contrast, fear of COVID-19 was significantly positively correlated with general anxiety disorder (r = 0.450, p < 0.01). These findings indicate that positive mental health could reduce fear of COVID-19 and general anxiety disorder, whereas fear of COVID-19 could increase general anxiety disorder among study respondents.

**Table 2:** Correlation matrix of study variables (n = 501)

Variables	PMH	FCV	PB	GAD
Positive mental health (PMH)	1	− 0.228**	0.122**	− 0.277**
Fear of COVID-19 (FCV)	–	1	0.328**	0.450**
Preventive behavior (PB)	–	–	1	0.132**
General anxiety disorder (GAD)	–	–	–	1

**Significant at the 0.01 level (2-tailed)

### Hierarchical linear regression

A hierarchical linear regression was conducted to indicate factors contributing to general anxiety disorder (Table 3). The findings of model 1 show that positive mental health reduces general anxiety among the respondents (β = − 0.277, R^2^ = 0.075, F = 41.602, p < 0.001). In model 2, fear of COVID-19 and preventive behavior was added along with positive mental health to predict general anxiety. The results indicate that positive mental health reduces general anxiety (β = − 0.189, p < 0.001), whereas fear of COVID-19 (β = 0.399, p < 0.001) increases it among the respondents (R^2^ = 0.236, F = 51.048, p < 0.001). However, preventive behavior does not explain the general anxiety among the respondents (β = 0.025, p = 0.561).

**Table 3:** Results of linear regression predicting general anxiety disorder (n = 501)

Model	Variables	B	SE	β	t	95% CI	p
1	(Constant)	19.944	1.503		13.268	16.990–22.897	< 0.001
	Positive mental health	− 0.275	0.043	− 0.277	− 6.450	− 0.358 to − 0.191	< 0.001
	R^2^ = 0.075, F = 41.602, p < 0.001						
2	(Constant)	10.232	1.694		6.039	6.903–13.562	< 0.001
	Positive mental health	− 0.187	0.041	− 0.189	− 4.584	− 0.268 to − 0.107	< 0.001
	Fear of COVID-19	0.331	0.036	0.399	9.211	0.260–0.401	< 0.001
	Preventive behavior	0.019	0.033	0.025	0.582	− 0.045 to 0.083	0.561
	R^2^ = 0.236, F = 51.048, p < 0.001						

SE, standard error; CI, confidence interval

### Structural equational modeling

Bearing in mind the results of the hierarchal linear regression, it can be concluded that positive mental health reduced general anxiety among the respondents. However, there is a need to investigate the role of positive mental health in controlling the fear of COVID-19. Moreover, correlational analysis indicates that there is a negative relationship between positive mental health and fear of COVID-19. To examine the interplay among positive mental health, fear of COVID-19 and general anxiety, we performed structural equational modeling by assuming positive mental health as an independent variable, general anxiety as a dependent variable and fear of COVID-19 as a mediating variable. The results show that all fit indices are within an acceptable range (χ2_(219, n=501)_ = 502.89, p < 0.05; RMSR = 0.046; RMSEA = 0.051; GFI = 0.921; CFI = 0.946).

**Fig. 1:**
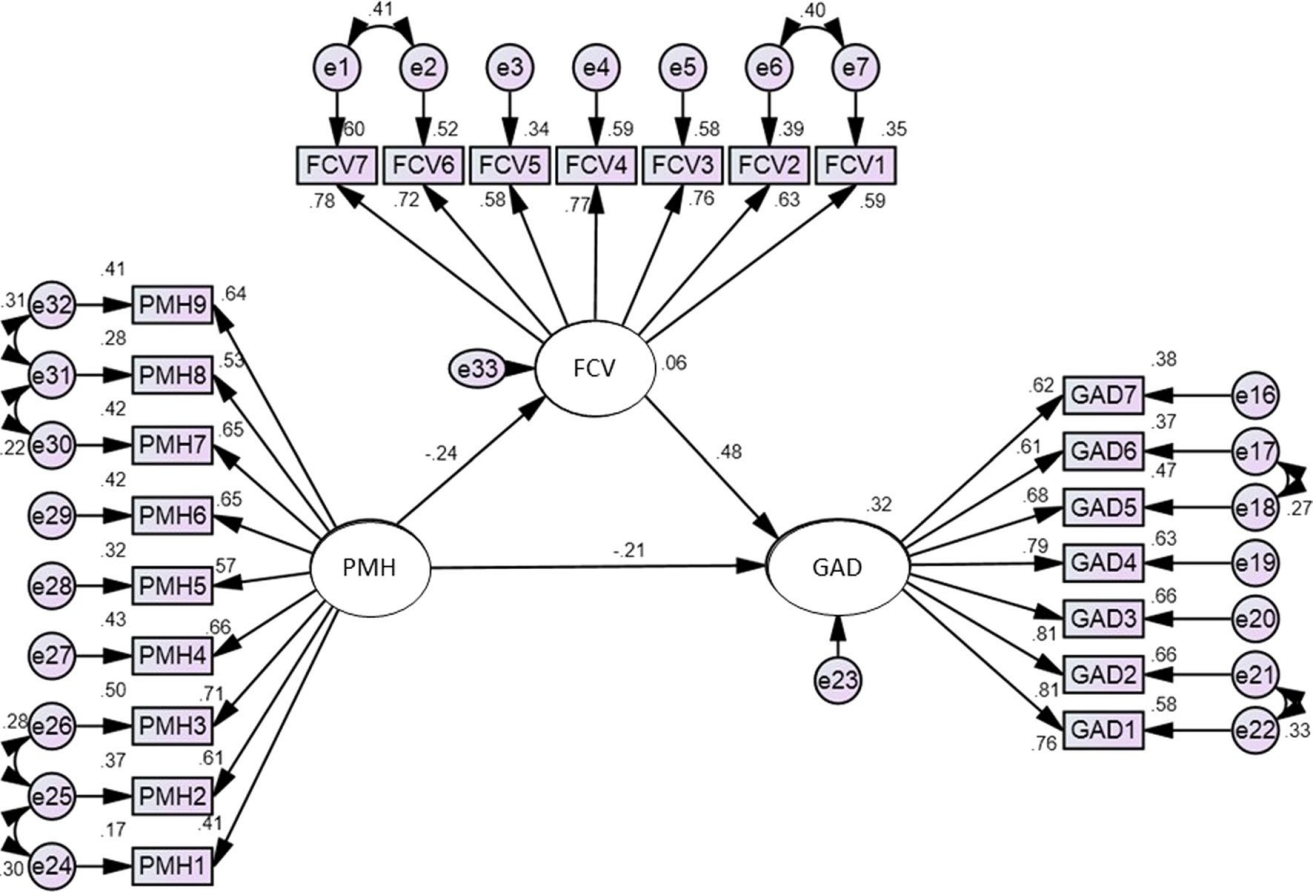
SEM model

Positive mental health had direct effects in reducing the fear of COVID-19 (β = − 0.244, p < 0.001) and general anxiety (β = − 0.210, p < 0.001). Fear of COVID-19 had a direct effect on increasing general anxiety (β = 0.480, p < 0.001). In addition to the direct effect of positive mental health on general anxiety, it also had an indirect effect (β = − 0.117, p < 0.001) via fear of COVID-19 on general anxiety. Therefore, it can be concluded that positive mental health reduces fear of COVID-19 and general anxiety among the respondents (Table 4, Fig. 1).

**Table 4:** Direct and indirect effects of positive mental health (n = 501)

Model	Direct effects (β)	Indirect effects (β)	Total effects (β)
PMH → FCV	− 0.244***		− 0.244***
PMH → GAD	− 0.210***	− 0.117***	− 0.327***
FCV → GAD	0.480***		0.480***

PMH, positive mental health; FCV, fear of COVID-19; GAD, generalized anxiety disorder

***Significant at the 0.001 level

## Discussion

To our knowledge, this is the first study investigating the role of positive mental health in reducing fear of COVID-19 and general anxiety disorder. It also examines the relationship between positive mental health and preventive behavior. The majority of studies related to mental health issues caused by COVID-19 have focused on negative mental health problems, such as anxiety [45], worry or panic [46], fear [15], feeling of worthlessness [47], boredom and irritability [48], social isolation [49], quarantine experience [50], sleeplessness [51], fear of infection [52], anger and cognitive decline [53], stress of obstructed healthcare [54], and feelings of loss or grief and stigma [55]. Studies on the importance of positive mental health in times of pandemics are scarce. The current study found high average scores of positive mental health and preventive behavior among the respondents.

With 220 million inhabitants, Pakistan is the sixth most populated country in the world. This large population is at high risk of the spread of COVID-19 and, consequently, high mortality, especially among the elderly and those with chronic diseases [56]. The anticipation of these risks, along with an infodemic, may potentially create fear and anxiety among the population. In the aftermath of the COVID-19 outbreak, fear has been observed among populations worldwide [13]. Fear can have devastating effects on individual’s mental health [6]. A mental health crisis can cause multiple obstructions to the effective management of the pandemic. A negative mental health status can substantially deteriorate an individual’s actions during panic situations, including preventive behavior. This study found moderate average scores of fear of COVID-19 and general anxiety disorder among the respondents.

The results show a significant negative relationship between positive mental health and fear of COVID-19 and general anxiety disorder. Positive mental health can be a protective factor against issues related to mental health [57]. A lack of positive mental health poses a substantial risk of depression and impaired physical health [58]. Positive mental health has shown a significantly positive association with preventive behavior. Large empirical evidence has reported that positive mental health and positive emotions broaden an individual’s awareness, encourage productive thoughts and actions, and promote caregiving behavior [59, 60]. It can be inferred that positive mental health stimulates preventive behavior among respondents.

Fear of COVID-19 has been positively and significantly correlated with general anxiety disorder. Anxiety and fear were predominantly found among COVID-19 patients in China [61]. Results of another study from Pakistan suggest that fear of COVID-19 is associated with anxiety sensitivity [62]. The results of the current study are also congruent with findings of an online-based survey conducted that found anxiety related to health issues and media use as predictors of fear of COVID-19 [63].

The findings from the hierarchical linear regression show that positive mental health reduces general anxiety, whereas fear of COVID-19 increases it. Moreover, preventive behavior does not explain general anxiety among the respondents. The results of the structural equational modeling also report that positive mental health reduces fear of COVID-19 and general anxiety.

The findings of this study suggest that when pandemic led people towards more anxiety, fear and stress lowering their happiness, positive mental health attitudes can foster optimal functioning and flourishing among individuals. It can enhance personal strength of people and can function as protective factor in face of health-related stress and anxiety. This study identified that utilizing strategies (e.g. to encourage sense of belongingness, appreciation, meaningfulness of work) to enhance positive mental health and contribute to the well-being of people suffering from anxiety and fear during the pandemic. These findings are extremely important with regard to (mental and physical) healthcare workers in order to understand their conditions. Media (print, electronic, and social) can be used to motivate people to take care of their mental health through positive activities, such as social interaction with loved ones, reading books or spiritual coping.

At the community level, social ties, community cohesion, hope, and a sense of collectivism and altruism can help communities to cope with pandemic driven depression and panic which can lead to positive outcomes. Future research could be conducted to examine the impact of positive mental health strategies in reducing fear and anxiety in diverse populations. Furthermore, cultural resources of positive mental health can be discovered. A positive mental health toolkit can be prepared in light of these findings.

### Limitations

One of the major limitations of this study refers to its cross-sectional design, which does not allow for causal interpretations. Furthermore, when interpreting the results, one need to keep in mind that the data have been collected in May 2020, representing the specific conditions at that time. The online-based data collection does not allow to calculate a response rate. In addition, due to the convenience sampling, the study cannot claim any representativeness. Also, the results may be valid to Pakistan—or at least the sample interviewed within Pakistan –, but this may be not the case for other countries. Therefore, this study highlights the need for investigating the impact of positive mental health to cope with COVID-19 from other cultural perspectives. Lastly, there may have been possible response bias by the respondents in answering the questions about positive mental health though researchers strove to ensure anonymity and confidentiality.

## Conclusion

In conclusion, the findings of this study revealed that general public in study has a good level of positive mental health and preventive behavior—despite a moderate level of fear of COVID-19 and anxiety. People with better positive mental health had less fear of COVID-19 and anxiety, while fear contributed to increasing anxiety. This study found that positive mental health enhanced the preventive behavior during the pandemic. Based on these findings, there is a need to develop community health policy emphasizing the devising of positive mental health strategies for people practicing social/physical distancing and isolation during pandemic. Appropriate mental health assistance focused on positive emotions must be provided to those experiencing fear and anxiety of pandemic. In context of growing concerns about prolongation of COVID-19, the findings of this study imply that positive emotions interventions can enhance wellbeing amid the wellbeing.

## Data Availability

The datasets used and/or analyzed during the current study available from the corresponding author on reasonable request. Due to the nature of this research, participants of this study did not agree to publish the disaggregated data publicly.

## Abbreviations

CFI: Comparative fit index
COVID-19: Coronavirus Disease 2019
FCV-19S: Fear of COVID-19 Scale
GAD: Generalized Anxiety Disorder
GFI: Goodness of fit
RMSEA: Root mean square error of approximation
RMSR: Root mean square residual
SD: Standard deviation
SEM: Structural equational modeling
WHO: World Health Organization
